# Surfactancy in a tadpole model of proteins

**DOI:** 10.1098/rsif.2022.0172

**Published:** 2022-10-05

**Authors:** O. T. Dyer, R. C. Ball

**Affiliations:** Department of Physics, University of Warwick, Coventry CV4 7AL, UK

**Keywords:** surfactant, depletion flocculation, liquid–liquid phase separation, intrinsically disordered region

## Abstract

We model the environment of eukaryotic nuclei by representing macromolecules by only their entropic properties, with globular molecules represented by spherical colloids and flexible molecules by polymers. We put particular focus on proteins with both globular and intrinsically disordered regions, which we represent with ‘tadpole’ constructed by grafting single polymers and colloids together. In Monte Carlo simulations, we find these tadpoles support phase separation via depletion flocculation, and demonstrate several surfactant behaviours, including being found preferentially at interfaces and forming micelles in single phase solution. Furthermore, the model parameters can be tuned to give a tadpole a preference for either bulk phase. However, we find entropy too weak to drive these behaviours by itself at likely biological concentrations.

## 1. Introduction

Liquid–liquid phase separation (LLPS) has been identified as a mechanism for eukaryotic cellsto organize their constituent biomolecules without needing the lipid bilayer membranes used by conventional organelles [1–3]. The resulting droplets are sometimes called ‘membraneless organelles’ [4]. The best and longest known example of these is the nucleolus, which is involved in the production of ribosomes [5], but more recent work has identified a variety of similar—albeit smaller—liquid aggregates inside both the nucleus and the cell cytoplasm [4]. Their locations, functions and compositions vary, but they frequently feature proteins with long sections of the polypeptide chain that remain flexible instead of folding into rigid secondary and tertiary structures [3,6–8]. These sections are called intrinsically disordered regions (IDRs) and can be found anywhere along the polypeptide chain. In this article, we will model IDRs at either the carboxy-terminal domain (CTD) or amino-terminal domain, leading to proteins with a flexible chain attached to a single globular region.

The role of these IDRs in LLPS remains uncertain, with most work to date focusing on weak attractive interactions between IDRs, especially those between charge dipoles, aromatic groups and net charges in amino acid residues [9]. The idea being that having longer IDRs provides more weak interactions with which to hold the droplet together.

These energetic considerations are not alone in driving phase behaviour, however, as entropy also plays a role. This has several contributions: entropy of mixing drives systems to mix, and thus hinders LLPS; conformational entropy of IDRs multiplies the number of microstates using the chemistry above [10]; and even without chemistry the conformational entropy introduces crowding effects that give IDRs an effective repulsion from globular molecules. Crowding effects, which are the focus of this work, are not new to cell biology. Studies have included the organization of DNA at different length scales [11–15], the influence of crowding on transcription rates [16] and on LLPS [11,17–20]. However, to our knowledge purely repulsive models of proteins with both IDRs and globular regions have not yet been studied.

Physics can provide insights into such systems by drawing analogy to polymer–colloid systems, which are characterized by the ratio of polymer radius of gyration and colloid radius *q* = *R*_*g*_/*R*_col_. Behaviour in the ‘colloid limit’, *q* ≪ 1, is very well established, with mixtures of non-adsorbing polymers and colloids separating via depletion flocculation [21–23]. The story in the ‘protein limit’, *q* > 1, is similar, but with the key difference that phase separation is less sensitive to *R*_*g*_ than the correlation length of the polymer mesh [24]. Recent work has argued similar mechanisms can play a role in biology where it can separate nuclear transport receptors in nuclear pores [25].

Interesting behaviour also arises when the polymers are grafted to proteins. In the colloid limit, it is common practice to coat the colloids with polymers, stabilizing them against the van der Waals forces driving flocculation by exploiting the entropic repulsion of the polymers [26]. In the protein limit, polymer–colloid grafts are often called grafted nanoparticles (GNPs), and have been studied extensively in the last two decades [27–34]. This work has focused largely on materials science, usually aiming to improve the properties of nanocomposites [32] or using specific interactions of the GNPs in the design of materials [33]. Research into GNPs with only one grafted polymer—an appropriate analogue to proteins with a single IDR—has been comparatively sparse [27–29,33].

Similarly to the biological LLPS literature, we are unaware of any work that has sought to quantify the importance of entropy in GNP behaviour. Hence, by modelling proteins with IDRs as single-chain GNPs with no attractive interactions, this article simultaneously fills gaps in both fields.

Finally, we recognize solvent entropy can also play a role via hydration effects and has been shown to be important in protein aggregation, even for proteins without IDRs [35–39]. In this work, we only consider explicitly the entropic properties of the macromolecules, with solvent entropy folded into net local solute interactions which we assume to be fully repulsive.

## 2. Methods

To study the role of IDRs’ entropy in LLPS, we run dynamic Monte Carlo simulations in continuous, three-dimensional, periodic boxes. The box dimensions are *L*_*x*_ × *L*_*y*_ × *L*_*z*_, where we always set *L*_*x*_ = *L*_*y*_ and take *L*_*z*_ as the box’s longest dimension. These dimensions will vary between sections, where they are stated.

We coarse grain macromolecules into collections of spheres where the *i*th particle has hard core radius *R*_*i*_. Since we focus solely on their entropic properties, these particles experience no attractions and repel each other with the hard sphere potential

2.1 $$U_{\mathrm{HS}}(r_{ij})=\left\{ \begin{array}{cc} & \;\infty \;r_{ij}<R_{i}+R_{\;j}\\ & \;0\;\mathrm{otherwise} \end{array}\right.$$

when particles *i* and *j* are distance *r*_*ij*_ apart.

**Figure 1.**
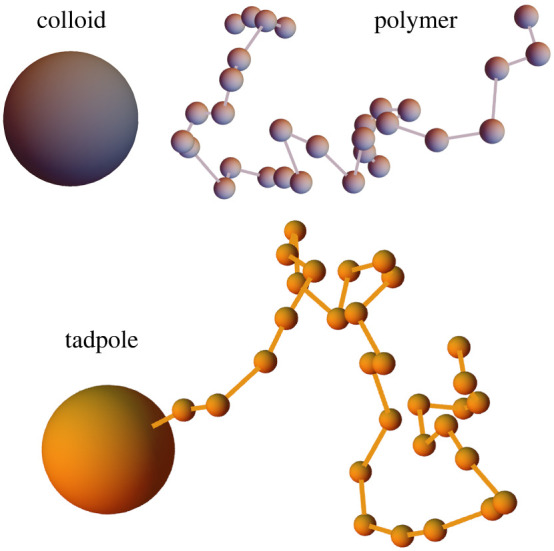
Examples of our model molecules. Note they are three-dimensional, so apparent overlaps are not real. The tadpole’s ‘head’ (large bead) is equivalent to a colloid, while the ‘tail’ is equivalent to a polymer. All beads are hard spheres and interact with potentials in equations (2.1) and (2.2).

Spherical particles are joined together into the three molecule species depicted in figure 1. Colloids are single large beads with radius *R*_col_ representing low-entropy (globular) macromolecules. Polymers are flexible, linear chains of *N*_mono_ small beads, each with radius *R*_mono_, that represent molecules with high conformational entropy. Finally, ‘tadpoles’ represent IDR-containing proteins with a globular region attached to a single IDR. These two regions are represented by a large ‘head’ bead with radius *R*_head_, and a ‘tail’ chain of *N*_tail_ small beads with radii *R*_tail_. To reduce the number of parameters in this work, we set *R*_head_ = *R*_col_ and *R*_tail_ = *R*_mono_, so a tadpole can be viewed as a colloid grafted to a polymer when *N*_tail_ = *N*_mono_. This helps minimize the number of parameters used in this work, but limits our ability to make quantitative comparisons with experiments.

All intra-molecular bonds are enforced with the hard square well potential

2.2 $$U_{\mathrm{bond}}(r_{i,i+1})=\left\{ \begin{array}{cc} 0 & r_{i,i+1}<R_{i}+R_{i+1}+3R_{\mathrm{tail}}\\ \infty & \mathrm{otherwise}\; \end{array}\right.,$$

which acts alongside *U*_HS_.

These molecules are placed in an implicit solvent and do not experience any hydrodynamic effects beyond their diffusivity being set by the usual Stokes–Einstein expression *D*_*i*_ = *k*_B_*T*/(6*πηR*_*i*_). Rather than specify the viscosity *η* explicitly, we define the time unit by the time taken for small particles to diffuse their own size, as listed in table 1. Similarly, we use the thermal energy *k*_B_*T* as our energy unit.

**Table 1.** Parameter values in this work, with code units specified in the top rows to help ground our results in their biological context. Where multiple values are used, their ranges are specified.

parameter	value in simulation
length unit, ℓ	12.5 nm
time unit, *τ*	$\pi \eta R_{\mathrm{tail}}^{3}/k_{B}T$
energy unit, *ɛ*	*k*_B_*T*
*R*_col_/ℓ, *R*_head_/ℓ	0.44
*R*_mono_/ℓ, *R*_tail_/ℓ	0.0908
*N*_col_	0–376
*N*_poly_	0–188
*N*_mono_	30
*N*_tad_	0–188
*N*_tail_	15–30
*δt*/*τ*	0.4

This diffusivity is achieved by displacing particles with the noise term used in the Brownian dynamics algorithm [40],

2.3 $$r_{i}(t+\delta t)=r_{i}(t)+\sqrt{2D_{i}\;\delta t}W,$$

where **W** is a Gaussian-distributed random vector with zero mean and unit variance in all three directions. The conservative interactions are implemented with a Metropolis acceptance test, passing with probability

2.4 $$P_{\mathrm{accept}}=\text{min}\left[1,\exp\left(-\frac{\Delta U}{k_{B}T}\right)\right].$$

With our hard systems, this means only accepting displacements if the particle’s final energy is zero. To maintain a high acceptance rate in this hard environment we set the time increment *δt* such that $\sqrt{2D_{i}\;\delta t}<R_{\mathrm{tail}}$.

The values of parameters used in this work are listed in table 1. Our choices for particle sizes lead to *q* ≈ 2.3 when *N*_tail_ = 30, which approximates the ratio for human RNA polymerase II’s globular region and the flexible CTD on its largest subunit [7,41].

Our criterion for equilibration is described in appendix A. Except where otherwise stated, our quantitative results average over the last 10 system configurations in each simulation, each separated in time by at least 3 × 10^4^*τ* to allow for local re-equilibration. We then further averaged over at least seven independent simulation runs for each system, giving us a minimum of 70 configurations contributing to each data point.

## 3. Entropic phase separation

Before showing the results of these simulations, it is helpful to explain the physics driving phase behaviour in our minimal model. By only considering hard interactions, all allowed states have the same energy and the temperature does not affect equilibrium states (although it does still play a role in dynamics). Free energy differences can, therefore, be written only in terms of the entropy:

3.1 $$dF=-T\;dS=-T\;dS_{m}-T\;dS_{c}.$$

Here, we have split the entropy of mixing (*S*_*m*_) and conformational entropy (*S*_*c*_), characterizing arrangements of molecule centres of mass and intra-molecule arrangements, respectively.

This split is relevant for crowding effects where flexible chains are obstructed by nearby rigid bodies, reducing the number of available conformations of the chain, and in turn leading to a significant reduction in *S*_*c*_. The archetypal system for this is a mixture of colloids and polymers, in which the two species can phase separate via depletion flocculation, where the dominance of *S*_*c*_ over *S*_*m*_ drives the colloids to cluster to minimize the contact of the two species, thus allowing the polymers to adopt the maximum number of conformations [21].

The expectation is that this remains significant in our systems with tadpoles present, and hence that proteins may have evolved IDRs to exploit it.

## 4. Results

### 4.1. Qualitative phase behaviour

**Figure 2.**
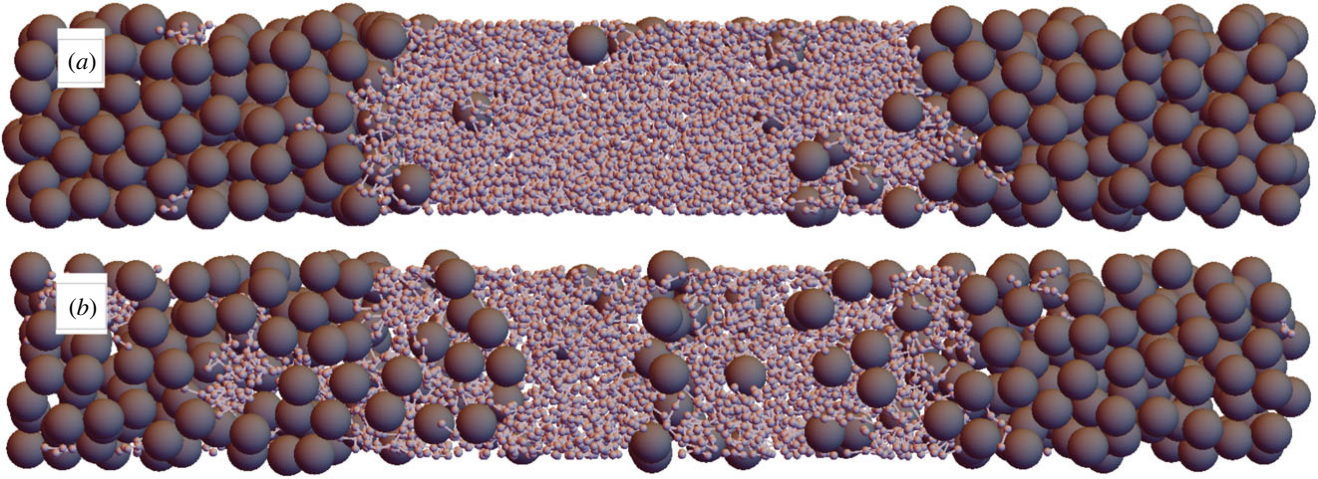
Snapshots of equilibrated polymer + colloid systems. Both systems have box dimensions 4 × 4 × 27. (*a*) *N*_col_ = 374, *N*_poly_ = 188. (*b*) *N*_col_ = 328, *N*_poly_ = 126.

We first confirm depletion flocculation occurs in our simulations by simulating systems with only polymers and colloids present. For this we used highly stretched periodic boxes, with dimensions 4 × 4 × 27, that produce well-defined planar interfaces perpendicular to the *z*-axis, and whose widths are much smaller than the bulk phases. The transverse dimensions of the box are large enough to prevent molecules interacting with their own periodic images, but are quite small to keep the computational cost of simulations manageable. Such systems are shown in figure 2, in which the denser system in figure 2*a* shows a clear separation into polymer- and colloid-rich phases, as expected. The interfaces between these phases are also predictably close to planar and perpendicular to the box’s long axis.

Reducing the system density allows for a greater concentration of the minority component in each phase, which is particularly notable by the number of colloids in the polymer-rich phase in figure 2*b*. Reducing the densities further ultimately reaches fully mixed states, and this will be analysed more quantitatively in §4.2.

Next, we add tadpoles to our systems, using the properties of our model molecules to make the replacement polymer + colloid → tadpole. In this way, the total number of large particles (colloids and tadpole heads) is kept constant, as is the number of small particles (in polymers and tadpole tails). This process can be viewed as the addition of *N*_tad_ bonds fusing polymers to colloids. Starting from the systems in figure 2, the concentrations are high enough that nonlinear terms in the osmotic pressure dominate, so the osmotic pressure is close to constant in each series of systems thus created, despite the number of molecules being reduced by *N*_tad_ from the initial polymer + colloid system.

**Figure 3.**
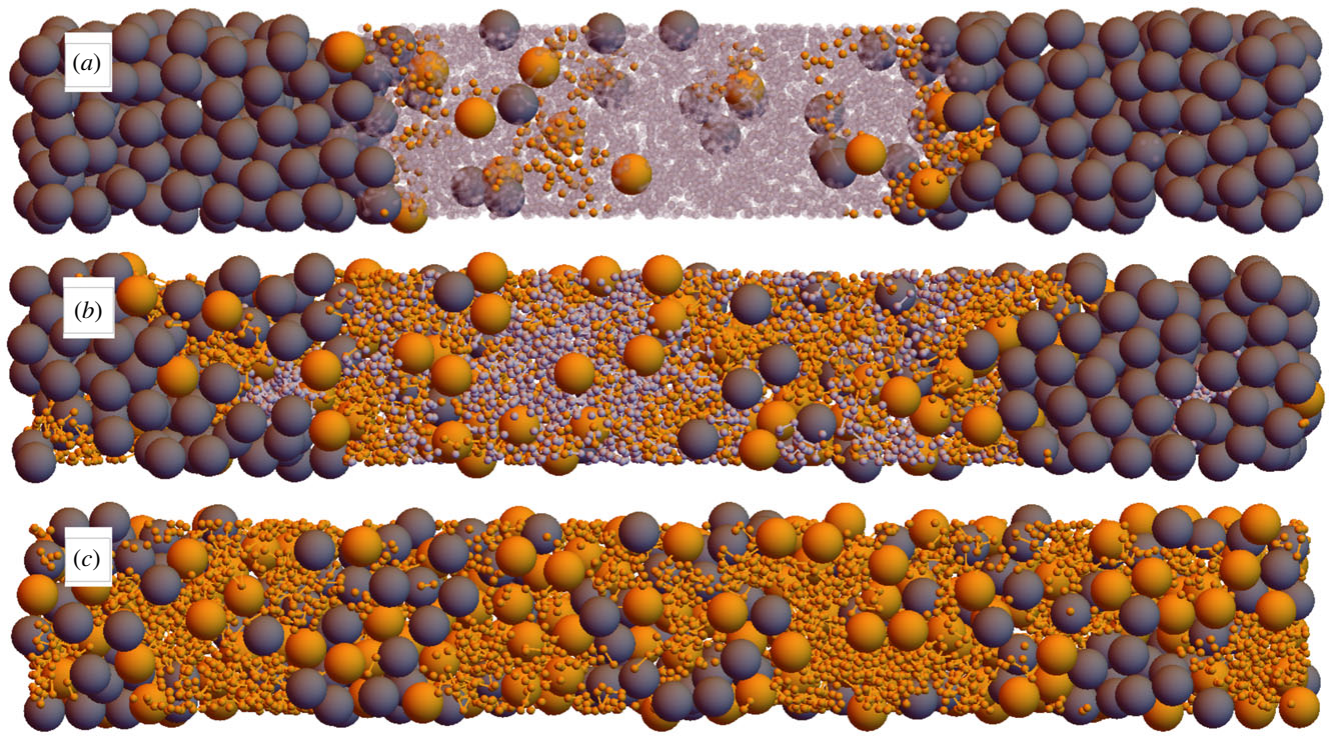
Snapshots of systems in the series replacing colloids and polymers with tadpoles (orange), starting from the system shown in figure 2*a*. The number of tadpoles added is (*a*) 14, (*b*) 94, (*c*) 188 (all polymers replaced). The opacity of polymers in (*a*) is reduced to improve the visibility of tadpoles in the polymer phase and at the interfaces. As in figure 2, the box dimensions are 4 × 4 × 27.

**Figure 4.**
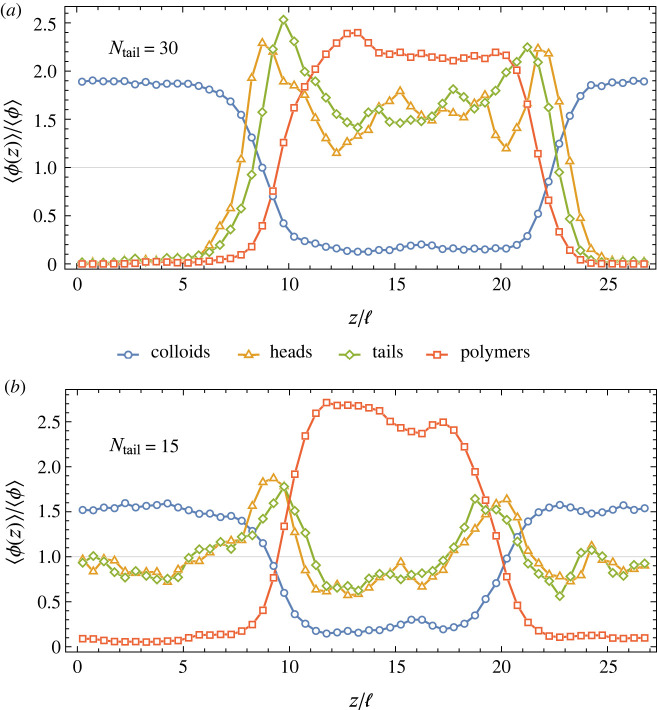
Plots of species volume fractions as a function of position along the simulation box, scaled by their global values. Both plots are taken from systems with *N*_col_ = 329, *N*_poly_ = 141 and *N*_tad_ = 47, with *N*_tail_ as indicated, and are averages over 80 simulation snapshots, each translated to align their interfaces.

Snapshots from such a series of simulations starting from figure 2*a* are shown in figure 3. Figure 3*a* demonstrates that adding a small number of tadpoles in this way has minimal impact on the volumes of the two bulk phases, and that of the two they prefer the polymer-rich phase. Figure 4*a*, which averages volume fractions in systems that have fused together 47 tadpoles, quantitatively shows the tadpole density drops to almost zero in the colloid-rich phase.

Furthermore, in figure 3*a* there appears to be a higher density of tadpoles at the interfaces than in the bulk. This would make intuitive sense as their heads and tails each prefer different phases when not bonded together, and hence one might expect tadpoles to want to straddle the interface to satisfy the preference of both their head and tail. This surfactant-like behaviour is confirmed quantitatively in figure 4*a*. Here, the tadpole density peaks at the interfaces, and close inspection finds the tadpoles tend to orient themselves so their heads and tails reside in the colloid and polymer phases, respectively, as seen by the peaks of the head density being closer to the colloid phase than the corresponding peaks in tail density.

When half of the polymers have been fused into tadpoles, shown in figure 3*b*, the two phases remain separated, but the polymer-rich phase expands to accommodate the additional volume of the tadpole heads. The interfaces have become more jagged, suggesting a reduction in interface tension (a point we will return to in §4.4), which is expected if the tadpoles are acting as surfactants. The tadpoles do not perfectly line the interfaces, however, which is indicative of the limited strength of the entropic ‘forces’ holding them there.

**Figure 5.**
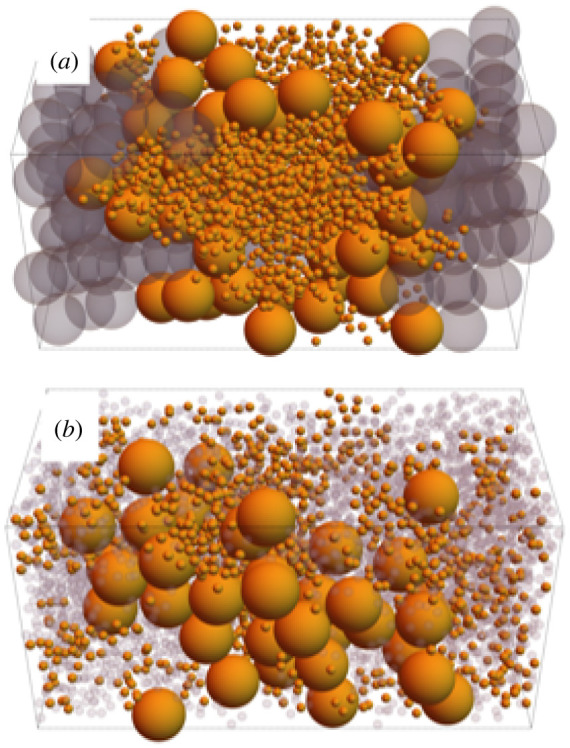
Example systems in which tadpoles form micelles (*a*) and inverted micelles (*b*). (*a*) tadpole + colloid system with *N*_tad_ = 56, *N*_col_ = 112. (*b*) tadpole + polymer system with *N*_tad_ = 42, *N*_poly_ = 126. The opacity of colloids and polymers has been reduced to help see the tadpoles. Both systems have box dimensions 4 × 4 × 8.

Adding yet more tadpoles, ultimately to the complete removal of polymers, yields the microphase separation seen in figure 3*c*. In this case, we find bicontinuous phases of tadpole tails and tadpole heads + colloids. By changing species concentrations, we can coax the tadpoles into forming micelles. Snapshots of these in shorter simulation boxes, 4 × 4 × 8, are shown in figure 5. In figure 5*a*, colloid density has been increased from the bicontinous phase, producing a cylindrical micelle with tadpole tails inwards, and whose axis is along the *x*-axis (front-to-back). For figure 5*b*, replacing colloids with polymers inverts the micelle so that the tadpole tails now point outwards, leaving a rod-like cluster of heads at its centre. These microphases are commonly produced by surfactant molecules [42], adding further evidence that tadpoles act as entropic surfactants.

### 4.2. Phase diagrams

**Figure 6.**
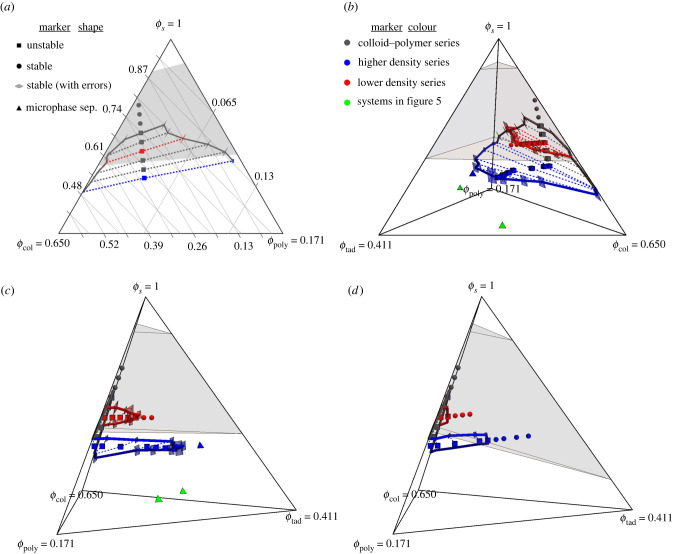
Phase diagrams for our model, with axes showing volume fractions for components—including the implicit solvent, *ϕ*_s_—such that they sum to 1 everywhere inside each diagram. To better see the binodal region, the low-*ϕ*_s_ part of the phase diagrams has been omitted, and polymer and tadpole axes are shown on different scales, leading to the non-equal volume fractions at the corners. The end points of the dashed tie lines within the data series have also been connected with lines of that series’ colour. The key by marker shape indicates the stability of different points, and errors at the ends of tie lines are shown as diamonds whose lengths in each direction are twice the standard error on the mean. The key by colour identifies different series of data, and both keys apply to all parts of the figure. The grey volume indicates the range of realistic osmotic pressures in [43], mapped into model concentrations as discussed in appendix B. (*a*) The ternary phase diagram for systems with *N*_tad_ = 0, where contours are labelled to help read the *ϕ* values of our data. (*b*) The three-dimensional phase diagram including tadpoles with *N*_tail_ = 30, with the two-dimensional diagram in (*a*) on its back-right face. (*c*) A different view of (*b*) showing how the binodals close as tadpoles are added. (*d*) The equivalent of (*c*) for systems with *N*_tail_ = 15.

We have constructed a phase diagram for our model in figure 6, using species volume fractions *ϕ* as axes. For the volume of each species, we sum the volumes of individual particles within them, multiplied by the number of those molecules present. Since the polymer-pervaded volume is more important than its bare volume fraction for phase behaviour, its volume fraction is shown on different scale from the colloid to improve the clarity of results; similar applies for the tadpoles. Note this shears the contours in our ternary plot, which are shown in figure 6*a*. Also, because our systems are compressible with varying total volume occupied by our particles, the implicit solvent needs including as its own component, hence including the solvent volume fraction *ϕ*_s_ = 1 − *ϕ*_col_ − *ϕ*_tad_ − *ϕ*_poly_.

Our primary motivation is to determine where these systems undergo macrophase separation, as is believed to happen inside the cell nucleus and cytoplasm. To that end, we have run several series of simulations as per the previous section, from which we map the binodal volume from the tie lines of macrophase-separating states. The end points of these tie lines were found by measuring the volume fractions in each bulk phase, and averaging this over our independent simulations. We can then compare this with the range of biologically relevant concentrations [43], shown as the shaded volume calculated using the statistical associating fluid theory (SAFT) formalism detailed in appendix B [44–47]. We use this formalism as a way to estimate the contribution of bonded chains in the pressure, which is difficult to measure in our hard systems, making it challenging to compare pressures directly. However, SAFT differs from our model in that it fixes bond lengths to exactly *R*_*i*_ + *R*_*j*_, and we found it over-predicts the number of our polymers required for a given pressure, as evidenced by the slightly different gradients of our tie lines and the shaded region’s lower edge in figure 6*a*. However, this will not alter our conclusions.

It is again helpful to focus first on the colloid + polymer systems with no tadpoles present. This face of the phase diagram is shown in figure 6*a*, in which the blue and red data, respectively, encompass the two systems shown in figure 2. This demonstrates quantitatively that as the density decreases (towards *ϕ*_s_ = 1), the number of colloids found in the polymer-rich phase increases, although the density of polymers found in the colloid-rich phase remains small until the systems are a low enough density to mix. Since such systems are nothing new [23], our main interest is in its overlap of its binodal and the shaded biological concentrations. While there is indeed some overlap, we caution that it is confined to the high concentration (low *ϕ*_s_) edge, and note that the author of [43] believed the lower osmotic pressures to be the most accurate. We are, therefore, mindful that the overlap we see may only exist due to inaccurate measurements extending the shaded region further than it should.

Whether that is the case or not, we infer from the limited overlap that entropy is unlikely to be strong enough by itself to drive biological phase separation. Nevertheless, it is strong enough that we believe it should not be neglected as a contributing factor, especially as it is independent of chemistry and thus part of the interaction in all systems.

Figure 6*b* shows the phase diagram produced when we add tadpoles as per §4.1. The higher density series of systems (in blue) includes those shown in figure 3 and extends out from the blue data in figure 6*a*. A second, lower-density series likewise extends out from the red data. This viewpoint is useful for visualizing how these series ‘grow’ the binodal out from the colloid + polymer diagram, ultimately closing when enough tadpoles are added in this way. Further viewpoints are provided in the electronic supplementary material to help picture this phase diagram in three-dimensional space.

The best viewpoint for seeing how quickly the binodal closes upon the addition of tadpoles is shown in figure 6*c*, where the tadpole volume fraction increases rightwards away from the colloid–polymer plane (now found at the left edge). Here, we see both series of simulations map out planes in the binodal that are viewed nearly edge-on and that run close to parallel to the lower boundary of the shaded volume. Interpolating the binodal between our series shows us that adding tadpoles decreases the overlap with the shaded volume. Hence, the addition of tadpoles does not help entropy drive phase separation, as compared with the polymer + colloid case. It, therefore, requires some inter-molecular attraction to account for biological phase separation.

Finally, we note that the micelle-producing systems in figure 5 were significantly more dense than biological systems (see the green triangles near the base of figure 6*c*). So while this is important behaviour for characterizing the general properties of our tadpoles, we do not expect it to have much biological relevance.

### 4.3. Differences when *N*_tail_ = 15

We now investigate what changes when we reduce the length of our tadpole tails from 30 to 15 beads, which would biologically represent shorter IDRs, e.g. the shorter CTDs of RNA polymerase II in yeast as compared with the human variant [7]. We keep the length of polymers unchanged, to represent an otherwise unchanged environment. Consequently, the replacement of colloids and polymers for an equal number of tadpoles no longer keeps the total number of beads fixed, and nor does the osmotic pressure remain approximately constant.

The phase diagram for these shorter tails is shown in figure 6*d*. In this, we show data series starting from the same two colloid + polymer systems as before, and again more viewpoints are provided in the electronic supplementary material. We kept the same axes as in the *N*_tail_ = 30 phase diagram to aid in their comparison, but due to the shorter tails, more tadpoles are required to create the same pressure. This is why the shaded volume now extends down to a larger value on the *ϕ*_tad_ axis. Our procedure for adding tadpoles now also *increases* *ϕ*_s_, leading to the data series rising as tadpoles are added. So, where the tie lines and the base of the shaded volume were nearly co-planar with *N*_tail_ = 30, they now pass through each other. On its own, this would help bring the binodal into the shaded volume.

However, we also find that the binodal now closes at a lower tadpole density for both data series. This shrinking of the binodal is the larger effect of the two, resulting in an overall reduction in the capacity for tadpoles with shorter tails to support phase separation, as would be expected from their smaller conformational entropy. We acknowledge, however, that longer tails also provide more interactions sites when the chemistry is accounted for, such that analogous differences in experiments cannot easily be attributed solely to differences in entropy.

Another major change with shorter tails is their shifting preferences for bulk phases. Where the *N*_tail_ = 30 tadpoles had a strong preference for the polymer-rich phase, figure 4*b* shows the concentration of tadpoles with *N*_tail_ = 15 is approximately equal in both bulk phases. Tail length can, therefore, be used to tune a macromolecule’s preference for the separating phases. It also has the advantage of being biologically accessible through mutations that repeat polypeptide sequences, as is thought to have happened with the CTD of RNA polymerase II, without changing chemical interactions in the process.

### 4.4. Interfacial tensions

We now seek to quantitatively confirm that the interfacial tension *γ* in our systems decreases with the addition of tadpoles. *γ* is difficult to access with hard potentials due to having infinitely poor statistics of the infinite forces present, so for this section we soften our system by using the Weeks–Chandler–Anderson (WCA) potential for particle repulsion,

4.1 $$U_{W}(s)=\left\{ \begin{array}{c} 4u_{W}\left(s^{-12}-s^{-6}+\frac{1}{4}\right)\;\mathrm{for}\;s<2^{1/6}\\ \;0\;\mathrm{otherwise} \end{array}\right.,$$

where *s* = *r*/*R*_W_ scales the particle separation, *r*, by the length scale of the WCA potential, *R*_W_. Bond potentials are also softened by using finitely extensible nonlinear elastic (FENE) bonds with energy

4.2 $$U_{F}(r)=\left\{ \begin{array}{cc} -\frac{1}{2}k_{F}R_{F}^{2}\ln\left[1-{\left(\frac{r}{R_{F}}\right)}^{2}\right] & \mathrm{for}\;r<R_{F}\\ \infty \; & \mathrm{otherwise} \end{array}\right..$$

The new parameters were set to *u*_W_ = *k*_B_*T* = *ɛ*, *R*_W_ = *R*_*i*_ + *R*_*j*_, *R*_F_ = *R*_*i*_ + *R*_*j*_ + 4*R*_tail_ between particles of species *i* and *j*. To match mean bond lengths to the hard systems, we set *k*_F_ = 7.822*ɛ*/ℓ^2^ between pairs of tail/polymer beads, and *k*_F_ = 2.558*ɛ*/ℓ^2^ between tadpole heads and tails.

With these potentials, we have well-defined forces that can be calculated from system configurations. This in turn lets us calculate the stress tensor σ and hence *γ* via the Kirkwood–Buff expression [48]

4.3 $$\gamma =-\frac{1}{2}\int_{0}^{L_{z}}\;dz\left(\sigma_{zz}-\frac{1}{2}(\sigma_{xx}+\sigma_{yy})\right),$$

where having two interfaces in the system contributes the front factor of 1/2.

Finally, we change the box dimensions to 6 × 6 × 12, which preserves the volume and hence particle numbers, but increases the ratio of interface area to bulk volume, improving our statistics. In these shorted boxes, we ran three sets of simulations equivalent to those in the higher density series in §4.1 fusing colloids and polymers into tadpoles with *N*_tail_ = 30. To improve our statistics, we ran these for longer to generate 200 independent snapshots, and in which we measured the interfacial tensions in table 2. These show a clear reduction in *γ* with the addition of tadpoles, as expected if they act as surfactants. These absolute values are in line with the commonly used estimate *γ* ≈ *k*_B_*T*/*δ*^2^, with *δ* being the interface width. We can also compare with the experimental values of 1–5 μNm^−1^ measured by Jawerth *et al.* [49], which overlaps with our range when we convert our units to SI, as shown in the fourth column of table 2.

**Table 2.** Interfacial tensions measured using equation (4.3) in our softened systems. Values in the third column have been scaled by the estimated interfacial width *δ* = 2*R*_col_(1 + *q*). In the fourth column, we have converted to SI units with *T* = 300 K, and *δ* = 36.3 nm.

*N*_tad_	*N*_tad_/*N*_poly_	*γ* *δ*^2^/*k*_B_*T*	*γ* (μNm^−1^)
0	0	9.2 ± 1.0	28.9 ± 3
47	1/3	4.1 ± 1.1	12.9 ± 3.5
94	1	0.4 ± 1.0	1.3 ± 3.1

## 5. Conclusion

We have used minimal, equilibrium Monte Carlo simulations of hard sphere systems to investigate the role of entropy in biological LLPS. By constructing phase diagrams for solutions of polymers, colloids and tadpole molecules representing biomolecules with different entropic properties, we were able to compare the concentrations at which such systems are unstable to macrophase separation with concentrations found in real cell nuclei. In doing so, we found entropy to be able to drive phase separation only at the upper estimates of biological concentrations. We conclude that the entropy from macromolecules is a significant contribution, but insufficient without some further attractions.

An important agenda for further work would be to assess the source, strength and impact of attractive macromolecular interactions, and whether that leaves them weak enough for IDRs to remain flexible. Within the implicit solvent paradigm, this requires inter-molecular interactions net of solvent effects, and the balance of exposed hydrophobic vs hydrophilic residues is a possible starting point. Beyond this, there are explicit solvent effects to be considered, including solvent entropy. The latter has been argued to be a significant driver of some LLPS, though its relevance where the solvent is not excluded from one phase is unclear [6,50,51].

Our simulations with tadpoles identified these molecules as entropic surfactants, exhibiting several typical surfactant behaviours: microphase separations such as micelles and bicontinuous phases; a preference for interfaces and a reduction in interfacial tensions as tadpoles are added. If such behaviour is present *in vivo*, it would be a major step forwards in understanding the biological importance of IDRs and their influence on liquid drop formation and structure. Indeed, if they exhibit strong segregation to the liquid drop interface, then the structure of ‘membraneless organelles’ might be closer to that of regular organelles than was previously realized.

Another avenue for further work could be to model IDR-containing proteins with different morphologies, such as a ‘pearl necklace’ model for IDRs connected to globular regions at both ends. We speculate that pearl necklace molecules would make less effective surfactants since both ends have low conformational entropy and would prefer the colloid-rich phase. However, this will be sensitive to the length of the IDR, which could tune the preference for the two phases and the interface like the length our tadpoles’ tails does.

Finally, in this article we simplified our model by matching particle sizes. There remains the task of exploring parameter space where particle sizes are not matched, which will be an important step towards obtaining quantitative results for comparison with the experiment.

## Figures and Tables

**Figure 7.**
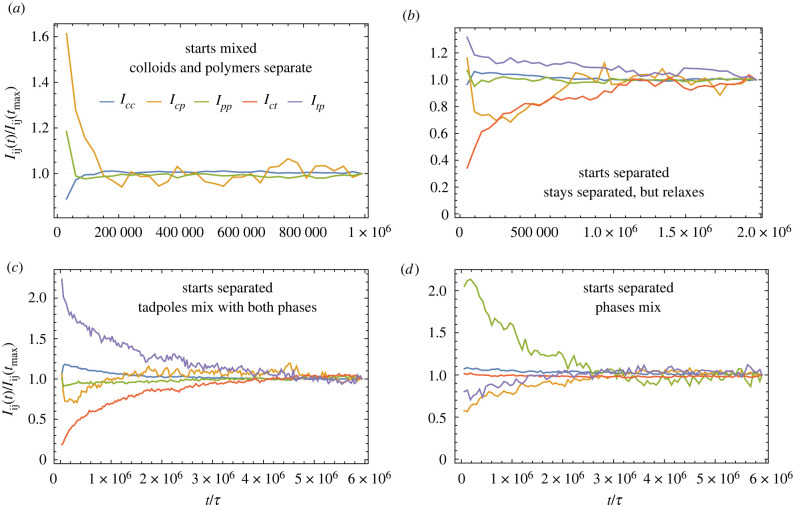
Plots of *I*_*ij*_(*t*), normalized by their value at the final time, showing the relaxation of cross-species radial distribution functions. The legend in (*a*) applies to all plots, and the subscripts correspond to c = colloid, p = polymer and *t* = tadpole species. The initial system in (*a*) was mixed, while the other systems were initialized as separated states. The numbers of molecules in each panel were: (*a*) *N*_col_ = 374, *N*_poly_ = 188, *N*_tad_ = 0; (*b*) and (*c*) *N*_col_ = 329, *N*_poly_ = 141, *N*_tad_ = 47; (*d*) *N*_col_ = 235, *N*_poly_ = 47, *N*_tad_ = 141. *N*_tail_ = 30 in (*a*) and (*b*), and *N*_tail_ = 15 in (*c*) and (*d*).

## Data Availability

The simulation and analysis codes can be found along with all data in the repository: http://wrap.warwick.ac.uk/163395/ The data are provided in the electronic supplementary material [52].

## Appendix A. Verifying equilibration

To determine when systems have equilibrated, we first calculate the radial distribution functions between all species *i* and *j*, at each time *t*: *g*_*ij*_(*r*, *t*). When doing this, we exclude pairs of particles within the same molecule.

We then integrate these like so

A1 $$I_{ij}(t)=\int_{0}^{2(R_{i}+R_{\;j})}\;dr\;r^{2}g_{ij}(r,t),$$

where the upper limit roughly corresponds to the radius of the first minimum in a typical liquid RDF. *I*_*ij*_(*t*) then quantifies the relaxation of the RDF. Figure 7 shows examples of this for a variety of systems evolving between different mixed and separated states. In all plots, we show *I*_cc_, *I*_pp_ and *I*_cp_ to show the relaxation of the two bulk phases. Where tadpoles are present, we also show *I*_ct_ and *I*_tp_ to characterize their relaxation with respect to both phases.

We used figure 7*a* to confirm our systems relax to separated states, and generated pre-separated states for systems containing tadpoles. In figure 7*b*,*c*, which corresponds to systems shown in figure 4, the tadpoles started in the polymer phase where they mostly stayed in figure 7*b* though they do prefer the interface leading to a notable increase in *I*_*ct*_ over time. In figure 7*c*, many tadpoles leave the polymer phase, leading to a larger increase in *I*_ct_ and decrease in *I*_tp_. The high densities made this a slow process, but exponential fits to our relaxation curves show we simulated out to about six times the relaxation time, making changes small enough at the end of the time series to treat as equilibrated.

In figure 7*d*, the tadpoles started with uniform density and the minority polymer component mixes with the colloid phase over time. This is seen by *I*_pp_ relaxing from a large value as polymers lose sight of each other.

## Appendix B. The SAFT formalism

SAFT provides a formalism for estimating the free energy in systems of associating particles, including polymers. Here, we summarize the SAFT expressions in [47], using our hard sphere potentials, starting by breaking the Helmholtz free energy into four terms

B1 $$F_{\mathrm{SAFT}}=F_{\mathrm{ideal}}+F_{\mathrm{part}}+F_{\mathrm{chain}}+F_{\mathrm{assoc}}.$$

These, respectively, represent the ideal gas term, the contribution from individual particles in the absence of bonds, the contribution from intra-chain interactions, and the contribution from inter-chain interactions.

For our hard systems *F*_assoc_ = 0, while the ideal gas term is the usual sum over molecule species

B2 $$F_{\mathrm{ideal}}=k_{B}T\sum_{i}N_{i}\left(\ln\left(\frac{N_{i}}{V\lambda_{\mathrm{th},i}^{3}}\right)-1\right),$$

with the system volume *V* and the thermal wavelength *λ*_th_.

The particle term uses an average contribution per particle calculated as

B3 $$\begin{array}{rl} \;f_{\mathrm{part}} & =\frac{1}{\zeta_{0}}\left(\left(\frac{\zeta_{2}^{3}}{\zeta_{3}^{2}}-\zeta_{0}\right)\ln(1-\zeta_{3})+\frac{3\zeta_{1}\zeta_{2}}{1-\zeta_{3}}+\frac{\zeta_{2}^{3}}{\zeta_{3}{\left(1-\zeta_{3}\right)}^{2}}\right), \end{array}$$

where

B4 $$\zeta_{\alpha }=\frac{\pi }{6V}\sum_{i}N_{i}\sigma_{i}^{\alpha }.$$

and *σ*_*i*_ is the diameter of particles of species *i*. Applying this to all particles gives

B5 $$F_{\mathrm{part}}=k_{B}T(N_{\mathrm{col}}+N_{\mathrm{tad}}(1+N_{\mathrm{tail}})+N_{\mathrm{poly}}N_{\mathrm{mono}})f_{\mathrm{part}}.$$

Finally, the chain term accounts for covalent bonds holding connected bead centres a distance (*σ*_*i*_ + *σ*_*j*_)/2 apart. I.e. the hard spheres are in contact. This is accounted for through the value of the radial distribution function at contact

B6 $$g_{ij}=\frac{1}{1-\zeta_{3}}+\frac{3B_{ij}}{{\left(1-\zeta_{3}\right)}^{2}}+\frac{2B_{ij}^{2}}{{\left(1-\zeta_{3}\right)}^{3}},$$

in which

B7 $$B_{ij}=\frac{\sigma_{i}\sigma_{\;j}}{\sigma_{i}+\sigma_{\;j}}\zeta_{2}.$$

Then

B8 $$\begin{array}{rl} F_{\mathrm{chain}} & =-k_{B}TN_{\mathrm{tad}}(\ln g_{\mathrm{ht}}+(N_{\mathrm{tail}}-1)\ln g_{\mathrm{tt}})\\ & \;\;-k_{B}TN_{\mathrm{poly}}(N_{\mathrm{mono}}-1)\ln g_{\mathrm{pp}}, \end{array}$$

counting all the bonds between tadpole heads, h; tadpole tail beads, t and polymer beads, p.

From the free energy expression, we get the (osmotic) pressure from *p*_SAFT_ = −(∂*F*_SAFT_/∂*V*)_*T*,*N*_. In this work, we solved this numerically to identify combinations of molecule densities with pressure equal to the upper and lower bounds found experimentally [43]. This range is then shown as the shaded volume in figure 6.
